# Programs Addressed to Family Caregivers/Informal Caregivers Needs: Systematic Review Protocol

**DOI:** 10.3390/jpm12020145

**Published:** 2022-01-21

**Authors:** Luís Sousa, Laurência Gemito, Rogério Ferreira, Lara Pinho, César Fonseca, Manuel Lopes

**Affiliations:** 1 São João de Deus School of Nursing, University of Évora, 7000-811 Évora, Portugal; mlpg@uevora.pt (L.G.); lmgp@uevora.pt (L.P.); cfonseca@uevora.pt (C.F.); mjl@uevora.pt (M.L.); 2 Comprehensive Health Research Centre (CHRC), 7000-811 Évora, Portugal; ferrinho.ferreira@ipbeja.pt; 3 School of Health of Beja, Polytechnic Institute of Beja, 7800-111 Beja, Portugal

**Keywords:** caregiver, informal caregiver, needs, intervention, patient-focused care

## Abstract

(1) Background: considering the growing increase in informal caregivers or family caregivers, it is critical to identify the unmet care needs of informal caregivers to improve their experiences, health, and well-being, contributing to the achievement of care needs of the elderly or people with adult dependency and promotion of successful transitions from health services to the community/home. (2) Objective: to identify the current state of knowledge about programs addressed to family caregivers/informal caregivers needs. (3) Methods: a systematic review will be undertaken with resource to databases from EBSCOhost Research Platform, Scopus, Web of Science, The Virtual Health Library (VHL). Studies published after January 2011 in English, Spanish, French, Italian and Portuguese will be considered. This review will consider all studies that report on any intervention program targeting family caregivers/informal caregivers who need to improve their experiences, health, and well-being, contributing to the meeting of their needs or those who have dementia and cognitive impairment, mental disorders, impairments in activities of daily living, frailty and/or who need health care and/or promoting successful transitions of community. (4) Discussion: The results of this review could be used to develop an intervention model to meet the needs of the family caregivers/informal caregivers. Furthermore, these findings will help to guide the construction of health policies regarding family caregivers/informal caregivers, as well their needs.

## 1. Introduction

The aging index of the European population is increasing rapidly, and a more significant increase is expected in the near future. Aging is related to health impairment and an increment in the number of chronic diseases [1]. This implies a significant global increase in the number of people living with chronic diseases [2]. Population aging is expected to increase the need for and consumption of long-term health care [1].

In this sense, many European countries have made significant reforms in the policy and systems of long-term care. In addition, cuts in professional health care are increasing the demand for informal care considerably, both by family and non-family caregivers [3,4].

Most people who need home care are older adults, have dementia and moderate cognitive impairment, dependence on activities of daily living, high degrees of fragility, and complexity in health care [5]. 

Most of these people remain at home, depending on informal caregivers to provide support to meet their needs [2]. Despite receiving awards associated with care, informal caregivers equally suffer from collateral effects, such as overload, stress, and physical and mental health problems [2]. In addition, family/informal caregivers of older adult residents in the community, specifically their children, reported a greater burden than other caregivers, even when referring to positive aspects in the provision of this care [6].

When older adults have dementia, the informal caregiver is the cornerstone and their suffering is associated with the worsening of various health outcomes of people with dementia, namely, worsening of the behavioral and psychological symptoms of dementia and abuse of the elderly [7]. 

Informal caregiver´s health suffers from the impact of caring, but its effect may vary according to the needs and expectations of informal caregivers and the support policies that are available to caregivers [4,8].

The causes of physical and psychological problems are related to four types of needs, mainly: (1) information on subjects related to mobility and prevention of falls, information on diseases, death, and dying; (2) social, financial, and e-health support; (3) organizational (balance between family and professional life, breaks, among others); and (4) social recognition [8]. When caring of people with cognitive disorders due to Alzheimer’s, the needs of the informal caregiver may evolve with the progression of the disease and the transition from dementia, and are related to information, psychosocial, social, psychoeducational support, among others. These needs should be assessed regularly and considered for the need for new knowledge, ability adjustment, support, and available services [9]. 

Currently, the support policies available in European countries such as Austria, Germany, Spain, France, Belgium, Czech Republic, Sweden, Netherlands, Denmark, Switzerland, Luxembourg, and Slovenia are the ones that promote better health for people cared for and for informal caregivers by: (1) providing off-duty time, (2) emotionally helping them to deal with care, and (3) teaching skill sets to improve care and deal effectively with these situations [4]. These intervention programs must be framed within a patient- and family-centered care perspective, as they take into account the patient’s values in personal care decisions, as well as including the patient´s and family’s role as essential counselors and partners in improving practices of care [10]. 

Patient- and family-centered care, as a set of interventions, aimed to meet the needs not only of the patient, but also of their families. Patient- and family-centered care is defined as mutually beneficial partnerships between healthcare professionals, patients, and families in the planning, delivery, and evaluation of healthcare [11]. Evidence indicates that patient- and family-centered care is considered a fundamental approach to improving the quality of health care through positive patient outcomes and generating a good working environment for healthcare professionals [12].

Bearing in mind the growing increase in informal family or paid caregivers, it is essential to identify the unmet care needs of the elderly and support needs for informal caregivers to improve their experiences, health, and well-being, contributing to achieve the care needs of the elderly and/or promoting successful transitions from health services to the community/home [6]. Knowledge of the unmet needs of informal/family caregivers allows to plan and provide more adjusted, patient- and family-centered care, with reduced gaps and higher quality [13]. 

### 1.1. Objective

The main objective of this review is to identify the current state of knowledge about programs addressed to family caregivers/informal caregivers needs.

### 1.2. Review Questions

This review aims to answer the following questions:

What are the strategies/interventions that address family caregivers/informal caregivers needs?

What are the health gains for the person being cared for, the informal caregiver/family caregivers, and the health system?

What are the facilitators and barriers identified in the implementation of interventions that address family caregivers/informal caregivers needs?

## 2. Materials and Methods

A systematic review of qualitative and quantitative studies was selected to be carried out, since it contributes to a better understanding of the most recent and available evidence on the topic; in addition, both perspectives are necessary to inform clinical policies or organizational decisions [14].

This protocol will follow the recommendations of the preferred reporting items for systematic reviews and meta-analyses (PRISMA) protocol statement [15].

The data from the included studies will be synthesized using a narrative synthesis approach and analyzed thematically to combine the results of studies that used several methods (qualitative, quantitative, and mixed methods).

This protocol was developed in February 2021 and the respective review will be completed by the end of 2021. PROSPERO registration number CRD42021241297.

### 2.1. Eligibility Criteria

This systematic review aims to ensure the accuracy and systematization proper to this type of study, and has established eligibility criteria which are presented below.

### 2.2. Population

Studies will be included whose populations are made up of informal caregivers/family caregivers of adults or elderly people, or who have dementia and cognitive impairment, mental disorders, activities of daily living with a disability, fragility and/or who need health care.

### 2.3. Types of Intervention(s)/Phenomena of Interest

This literature review will consider studies on any family-centered care program addressed to family caregivers/informal caregivers needs in order to improve their experiences, health, and well-being, contributing to meeting the needs of adults (>18) or elderly people, or who have dementia and cognitive impairment, mental disorders, activities of daily living disability, fragility and/or who need health care and/or promoting successful transitions of community/home health services.

In a Portuguese study, the most prevalent health needs found were food preparation, medication/taking tablets, taking care of the house, using the bathroom, sensory problems, communication/interaction, bladder, intestines, eating and drinking, memory, sleeping, and preventing falls [16].

Some of the interventions targeting informal caregivers are based on the recognition and application of anxiety management interventions, as well as depression and caregiver burden [17]. This includes individualized and high quality interventions, such as psychological and emotional stability [16], and interventions to preserve and enhance social support as a way to reduce depressive symptoms of informal caregivers [18].

### 2.4. Comparison

This review will include studies with or without a comparative group.

### 2.5. Phenomena of Interest

The qualitative component of this review will explore the experiences of caregivers who have participated in intervention programs, and the facilitators and barriers identified in the implementation of these programs.

### 2.6. Study Design

This systematic review should include primary empirical studies. The quantitative component of the review will consider both experimental and epidemiological study designs, including randomized controlled trials, non-randomized controlled trials, quasi experimental, before and after studies, prospective and retrospective cohort studies, case–control studies, and analytical cross-sectional studies.

The qualitative component of the review will contemplate studies that focus on qualitative data including, but not limited to, designs such as phenomenology, grounded theory, ethnography, and action research.

### 2.7. Context

All studies on programs that address the unmet care needs of informal caregivers/family caregivers of community-dwelling adults or elderly people, or who have dementia and cognitive impairment, mental disorders, activities of daily living with a disability, fragility and/or who need health care, regardless of context (community, culture, or specific environment), will be included in this review. Studies carried out in European countries will be included. 

### 2.8. Primary Outcome

The main outcomes appraised would be indicators of worsening health, well-being and financial; these can be assessed in a general or specific way when applied to informal caregivers/family caregivers of dependent people in the community. The data obtained may be represented quantitatively, such as averages, measures of prevalence or incidence, frequencies, economic indicators concerning evaluations, or they may be of a qualitative essence.

With the analysis of quantitative studies, we expect to find results that incorporate psychosocial or satisfaction indicators, such as stress, anxiety, depression, quality of life, family functioning, family empowerment, or satisfaction with family-centered care.

### 2.9. Secondary Outcomes

The secondary outcomes considered in this review will be the facilitators and barriers identified in the implementation of patient- and family-centered care programs that address the unmet care needs informal caregivers/family caregivers of community-dwelling dependent people. 

### 2.10. Search Strategy

#### 2.10.1. Data Sources

Given the nature of the research, the strategy to be adopted consists of the conduction of a comprehensive bibliographic search with recourse to the following databases: EBSCOhost Research Platform (CINAHL^®^ Plus with Full Text; Nursing & Allied Health Collection; Cochrane Plus Collection, including Cochrane Central Register of Controlled Trials; Cochrane Database of Systematic Reviews (CDSR) e Database of Abstracts of Reviews of Effects (DARE); MedicLatina; MEDLINE^®^, including International Nursing Index). PubMed via MEDLINE, CINAHL, Scopus, Web of Science, and The Virtual Health Library (VHL). Open Grey e Grey Literature Report.

#### 2.10.2. Search Terms

Subject headings (MeSH) will be used and according to it, an arrangement of four key concepts will include: Caregiver, Patient-Centered Care, Disabled Persons, Community-Based Distribution in the title, keywords, and abstract. Free terms such as program, intervention, and approach may be used. In this case, the search will be: ((Caregiver) OR (Care Giver*) OR (Caregiver*, Family) OR (Caregiver*, Spouse)) AND ((Disabled Persons) OR (elderly dependent) OR (Aged) OR (dementia) OR (Alzheimer) OR (Disability) AND ((Care, Non-Professional Home) OR (Nonprofessional Home Care) OR (Old Age Assistance) OR (Patient-Centered Care) OR (Patient Centered Nursing) OR (Patient-Focused Care) OR (needs assessment) OR (care management) OR (health care) OR (psychosocial care) OR (community) OR (Community-Based Distribution).

The strategy will be adapted according to each database and will be restricted to the last 10 years, therefore, from 2011 to 2021, in English, Portuguese, Spanish, French, and Italian. The pre-test of the research is found in Table 1.

The studies resulting from the research in each database will be exported to Microsoft Word^®^ (Microsoft, Redmond, WA, USA) and duplicates will be removed.

### 2.11. Data Collection and Analysis

#### 2.11.1. Selection of Studies

The criteria selected for the inclusion of studies in this investigation will consist of the reading of title, abstract, and keywords by two reviewers, and those which do not meet the inclusion criteria established for this review will not be included in it. A third reviewer will be included in case of divergences or doubts. Subsequently, the full texts will also be evaluated by two reviewers independently. For the presentation of this selection process, the PRISMA flowchart, with the results of the screening in the different phases, will be presented.

#### 2.11.2. Quality Appraisal

Prior to the inclusion of any component in this review, the quantitative articles selected from retrieval will be examined by two independent reviewers to validate them methodologically using standardized critical appraisal tools of the Joanna Briggs Institute Review and Statistical Evaluation of Meta-Analysis Instrument (JBI-MAStARI) to validate its inclusion in the review. 

Should discrepancies exist between the reviewers’ assessments, these will be solved through discussion, or by the intervention of a third reviewer.

The qualitative articles selected from retrieval will be examined by two independent reviewers in order to validate them methodologically using standardized critical appraisal tools of the Joanna Briggs Institute Qualitative Assessment and Review Instrument (JBI-QARI) to validate its inclusion in the review. 

Should discrepancies exist between the reviewers’ assessments, these will be solved through discussion, or by the intervention of a third reviewer. 

The process of results exposure of the critical appraisal will consist of a narrative form and in a table.

Regardless of the outcome of the methodological quality assessment, all studies will be part of the data synthesis phases of the review.

#### 2.11.3. Data Extraction

With regard to data extraction, at the initial stage, an individual descriptive analysis of each case will be carried out, considering the new research questions, and using extraction tools designed for the purpose.

Using JBI-MAStARI data extraction tools, quantitative data will be extracted from the articles to be included in the analysis.

Exhaustive details of the data extracted will be set out, including the purpose of the study, participants, assessment tools, intervention, significant outcomes and key findings, and study design. Standardized JBI data extraction tools will be used to extract data from the articles to be included in the review and to identify facilitators and barriers associated with program implementation.

Two independent reviewers will perform the extraction of the data and should discrepancies exist between the reviewers’ assessments, these will be solved by the intervention of a third reviewer. 

### 2.12. Strategy for Data Synthesis

The intention is to synthesize the results and present them using a table and the help of text and figures.

Whenever possible, the qualitative research findings will be gathered with the meta-aggregation approach. This will involve aggregating or synthesizing the findings to generate a set of statements that represent that same aggregation, bringing the findings together and categorizing them based on similarity of meaning.

These categories will then be synthesized to produce a range of integrated conclusions than can be used as a standard for evidence-based practice.

The narrative form of presenting results will be considered whenever the textual pooling is not feasible.

### 2.13. Patient and Public Involvement

During the construction or development of this review, there will be no involvement of patients or persons.

### 2.14. Ethics and Dissemination

Only secondary data will be considered and, therefore, no ethical approval is required to implement this study. This study consists of a systematic review protocol, that being, the results have not been extracted or analyzed yet, and will only be published through a publication after peer-review. 

## 3. Discussion

Caregivers of adults with dementia or disabilities presented as the largest number of the whole group of caregivers. These caregivers, like caregivers of people with dementia or disability, delivered a broad spectrum of health-related tasks and experienced negative emotions, including mainly anxiety, depression, caregiver burden, and restrictions on social participation [17,18,19,20].

The unmet needs of informal caregivers may be one of the main areas for improvement in policy and service delivery [21]. Although the circumstances and conditions of informal caregivers/family caregivers have generally improved in recent years, longitudinal monitoring in family care in old age is extremely important [22].

With this review, we intend to summarize the main intervention programs to address the needs of family/informal caregivers, not only for those identified as high risk, but also those at medium and low risk. In this sense, the identification and implementation of interventions focused on informal caregivers/family caregivers of community-dwelling adults or the elderly, or who have dementia and cognitive impairment, mental disorders, impairment of activities of daily living, fragility and/or who need health care, must reduce anxiety, depression, and caregiver burden, prevent disease conditions, and improve quality of life.

The programs and services most often offered to caregivers are dementia management education and training services through the course of work, specific psychosocial support service, respite services, and information or advice on legal rights issues. In the context of the coronavirus disease 2019 (COVID-19), many caregivers experience an increase in social isolation, a greater burden of care, and deteriorating physical and mental health. Programs such as the World Health Organization iSupport, mDementia, and e-mhGAP (Mental Health Gap Action Programme) play an important role in supporting the caregiver, as they provide the opportunity to overcome obstacles related to access and care, as well as the interruption of services due to COVID-19 [23].

## 4. Strengths and Limitations of This Study

This review protocol may have important implications for teaching in the health field, especially in nursing education, because if family care is almost absent from nursing education curricula and practice standards, nurses will not have the knowledge needed to enhance the role of family caregivers in patient-centered care teams and will not be able to meet the health needs of caregivers themselves.

The results of this review could be used to develop an intervention model to answer the needs of the informal caregiver. These findings will also help to guide the construction of health policies regarding informal caregivers taking in account their needs.

Regarding the limitations of this study, the research strategy will be adapted according to each data base and will be restricted to the last 10 years, i.e., from 2011 to 2021 in the English, Spanish, French, Italian, and Portuguese languages. 

## Figures and Tables

**Table 1:** Search Strategy.

Data Base	Search Strategy
CINAHL^®^ Plus with Full Text Nursing & Allied Health Collection; Cochrane Central Register of Controlled Trials; Cochrane Database of Systematic Reviews; Cochrane Methodology Register; Cochrane Clinical Answers; MedicLatina; MEDLINE^®^ with Full Text	(((Caregiver) OR (Care Giver*) OR (Caregiver*, Family) OR (Caregiver*, Spouse)) AND ((Disabled Persons) OR (elderly dependent) OR (Aged) OR (dementia) OR (Alzheimer) OR (Disability)) AND ((Care, Non-Professional Home) OR (Nonprofessional Home Care) OR (Old Age Assistance) OR (Patient-Centered Care) OR (Patient Centered Nursing) OR (Patient-Focused Care) OR (needs assessment) OR (care management) OR (health care) OR (psychosocial care) OR (community) OR (Community-Based Distribution)))
Scopus	TITLE-ABS-KEY ((caregiver*) AND ((disabled AND persons) OR (elderly AND dependent) OR (aged) OR (dementia) OR (Alzheimer) OR (disability)) AND ((nonprofessional AND home AND care) OR (patient-centered AND care )))
Web of Science	3#-660-#2 OR #1 Bases de dados = WOS, CCC, DIIDW, KJD, MEDLINE, RSCI, SCIELO Tempo estipulado = 2011–2021 Idioma da pesquisa = Auto 2#-400–KP = (((Caregiver) OR (Care Giver*) OR (Caregiver*, Family) OR (Caregiver*, Spouse)) AND ((Disabled Persons) OR (elderly dependent) OR (Aged) OR (dementia) OR (Disability)) AND ((Care, Non-Professional Home) OR (Nonprofessional Home Care) OR (Old Age Assistance) OR (Patient-Centered Care) OR (Patient Centered Nursing) OR (Patient-Focused Care) OR (needs assessment) OR (care management) OR (health care) OR (psychosocial care) OR (community) OR (Community-Based Distribution))) Bases de dados = WOS, CCC, DIIDW, KJD, MEDLINE, RSCI, SCIELO Tempo estipulado = 2011–2021 Idioma da pesquisa = Auto 1#-261–TI = (((Caregiver) OR (Care Giver*) OR (Caregiver*, Family) OR (Caregiver*, Spouse)) AND ((Disabled Persons) OR (elderly dependent) OR (Aged) OR (dementia) OR (Disability)) AND ((Care, Non-Professional Home) OR (Nonprofessional Home Care) OR (Old Age Assistance) OR (Patient-Centered Care) OR (Patient Centered Nursing) OR (Patient-Focused Care) OR (needs assessment) OR (care management) OR (health care) OR (psychosocial care) OR (community) OR (Community-Based Distribution))) Bases de dados = WOS, CCC, DIIDW, KJD, MEDLINE, RSCI, SCIELO Tempo estipulado = 2011–2021 Idioma da pesquisa = Auto
The Virtual Health Library (VHL)—abstract (MEDLINE; LILACS; IBECS)	(((Caregiver) OR (Care Giver*) OR (Caregiver*, Family) OR (Caregiver*, Spouse)) AND ((Disabled Persons) OR (elderly dependent) OR (Aged) OR (dementia) OR (Alzheimer) OR (Disability)) AND ((Care, Non-Professional Home) OR (Nonprofessional Home Care) OR (Old Age Assistance) OR (Patient-Centered Care) OR (Patient Centered Nursing) OR (Patient-Focused Care) OR (needs assessment) OR (care management) OR (health care) OR (psychosocial care) OR (community) OR (Community-Based Distribution)))

## Data Availability

Not applicable.
